# CFD-Guided Shadowing-Aware Acoustic Path Selection for Accurate Wind Estimation in Ultrasonic Anemometers

**DOI:** 10.3390/s26144488

**Published:** 2026-07-15

**Authors:** Tien Minh Khoi Nguyen, Tan Dung Nguyen, Le The Anh Vi, Thanh Dat Le, Thanh Dam Nguyen, Minh Quan Nguyen, Jae Sung Ahn, Tan Tien Nguyen, Sudip Mondal, Vu Hoang Minh Doan, Jaeyeop Choi, Junghwan Oh

**Affiliations:** 1 Industry 4.0 Convergence Bionics Engineering, Pukyong National University, Busan 48513, Republic of Korea; ntmkhoi@pukyong.ac.kr (T.M.K.N.); viletheanh0511@gmail.com (L.T.A.V.); dat.lt198640@gmail.com (T.D.L.); nguyendam5555@gmail.com (T.D.N.); quannguyenn1012@gmail.com (M.Q.N.); 2 Faculty of Engineering, Vietnamese-German University, Ho Chi Minh City 700000, Vietnam; dung.nt2@vgu.edu.vn; 3 Smart Gym-Based Translational Research Center for Active Senior’s Healthcare, Pukyong National University, Busan 48513, Republic of Korea; jsahn@pknu.ac.kr; 4 Key Laboratory of Digital Control and System Engineering (DCSELab), Faculty of Mechanical Engineering, Ho Chi Minh City University of Technology (HCMUT), Ho Chi Minh City 700000, Vietnam; nttien@hcmut.edu.vn; 5 Digital Healthcare Research Center, Institute of Information Technology and Convergence, Pukyong National University, Busan 48513, Republic of Korea; smondal@pknu.ac.kr; 6 Ohlabs Corp., Busan 48513, Republic of Korea

**Keywords:** ultrasonic anemometer, wind measurement, transducer shadowing, acoustic propagation, computational fluid dynamics

## Abstract

Ultrasonic anemometers are widely used for wind measurement owing to their fast response, high temporal resolution, and long operational lifetime with minimal recalibration requirements. However, most configurations suffer from transducer shadowing, where cylindrical probes disrupt local airflow and introduce systematic errors in time-of-flight (TOF) measurements. While prior computational fluid dynamics (CFD)-based investigations have characterized this effect, their analyses remain confined to low wind speeds, and no existing study has explicitly proposed a method to mitigate shadowing-induced bias. This paper presents a coupled CFD and acoustic propagation framework to analyze wake-induced velocity deficits and their effects on TOF measurements for a three-transducer ultrasonic anemometer, with simulations spanning 5 to 75 m/s over the full 360° range. The results show that shadowing distortions are strongly direction-dependent, peaked within approximately ±5° angular sectors, with a near-constant velocity deficit of approximately 40% along affected paths. A shadowing-aware acoustic path selection method is then proposed that selectively excludes corrupted acoustic paths, reducing the average velocity root-mean-square error (RMSE) to 0.349 m/s and the directional RMSE to 1.14°, representing improvements of more than an order of magnitude over shadow-unaware methods. These findings provide a physically grounded, simulation-based framework for shadowing-aware wind measurement using ultrasonic anemometers.

## 1. Introduction

Wind velocity and direction measurement plays a fundamental role across a wide range of applications, ranging from meteorological field monitoring [1,2,3] to unmanned aerial vehicle (UAV) platforms, where the wind vector can be inferred indirectly from vehicle dynamics [4,5] or measured directly using onboard ultrasonic anemometers [6,7,8]. Conventional wind sensors such as cup anemometers rely on mechanical components, which are susceptible to friction, inertia, and wear over time, limiting their suitability for long-term deployments [9,10]. Thermal anemometers offer improved temporal resolution but remain sensitive to temperature fluctuations and require frequent calibration [11]. In contrast, ultrasonic anemometers offer a compelling combination of high temporal resolution, absence of moving parts, simultaneous measurement of wind speed and direction, and low maintenance requirements, making them increasingly attractive for compact and real-time wind sensing applications [12,13,14,15].

Ultrasonic anemometers operate by measuring the time-of-flight (TOF) of acoustic pulses propagating between pairs of transducers, from which the wind velocity component along each acoustic path is extracted [16]. Alternative measurement principles include the ultrasonic resonance wind sensing (URWS) method, which exploits resonance frequency shifts induced by airflow [17], and cross-correlation approaches that estimate propagation delay from signal waveform matching [18]. Among these, bidirectional TOF remains the most widely adopted principle due to its direct physical interpretation, temperature self-compensation through differential measurement, and compatibility with digital signal processing. The sensor configuration determines the number of independent wind projections available for vector reconstruction: two-transducer systems yield a single path and cannot resolve wind direction without additional assumptions [19,20]; four- and six-transducer configurations provide redundancy and three-dimensional measurement capability but at increased hardware complexity and cost [8,21]. Three-transducer arrangements provide three independent velocity components in a planar configuration sufficient for two-dimensional wind vector reconstruction with inherent measurement redundancy [17].

Accurate estimation of flow-induced measurement errors requires a detailed understanding of the aerodynamic interactions around the sensor structure. In this context, computational fluid dynamics (CFD) has become a powerful tool for analyzing flow distortion around ultrasonic anemometer geometries. Zdravkovich [22] and Williamson et al. [23] provided foundational analyses of the flow around circular cylinders, establishing the wake formation mechanisms relevant to transducer geometries.

Building on these aerodynamic foundations, several studies have applied CFD to characterize transducer shadowing in ultrasonic anemometers. Chen et al. [19] employed CFD to quantitatively characterize the shadowing effect in a conventional two-transducer configuration, demonstrating through velocity nephograms and vorticity maps that transducer geometry introduces non-negligible, direction-dependent flow distortions; however, their analysis was limited to wind speeds of approximately 13.45m/s or less. Li et al. [24] further extended this line of investigation by combining Particle Image Velocimetry (PIV) with CFD simulations for a reflective ultrasonic anemometer, revealing that recirculation vortices downstream of upstream transducers produce path-averaged velocity errors with average relative deviations of approximately 3.38% and peak errors reaching 4.25%. Yet, their study similarly remained confined to a wind speed of 20m/s. Zhao et al. [18] proposed an adaptive cross-correlation algorithm for wind speed and direction estimation; however, the absence of CFD-based aerodynamic modeling in their work means that shadowing-induced bias was not captured, and the reported performance does not reflect physically realistic flow conditions. Together, these studies demonstrate that the extent and structure of wake regions are primarily governed by transducer geometry and the wind incidence angle [25]. Yet, a systematic characterization of shadowing behavior across the full operational velocity range remains absent.

The foregoing review identifies three principal research gaps. First, existing CFD-based shadowing studies have been confined to relatively low wind velocity ranges, which do not reflect the full operational envelope of ultrasonic anemometers deployed for meteorological or UAV applications, where wind speeds can substantially exceed these limits. As a consequence, the progressive deterioration in measurement accuracy at higher wind speeds due to stronger wake interactions remains uncharacterized. Second, estimation-oriented studies of three-transducer configurations have not explicitly incorporated CFD-based aerodynamic modeling, leading to performance evaluations that do not account for the bias introduced by transducer shadowing under realistic flow conditions. Third, and most critically, few studies have explicitly proposed a shadowing-aware acoustic path-selection method that identifies and excludes wake-corrupted acoustic paths during operation. Consequently, the combined effects of aerodynamic distortion and acoustic propagation on ultrasonic wind measurements remain insufficiently characterized in three-transducer configurations.

To address these gaps, this paper makes the following contributions:

- A coupled CFD and acoustic propagation simulation framework, implemented in COMSOL Multiphysics 6.2, is developed to characterize wake-induced velocity deficits and their effect on TOF measurements across a wide operational wind speed range.
- The directional and velocity dependence of the shadowing effect is systematically analyzed across the full $360^{\circ }$ range, providing quantitative physical insight into the governing mechanisms of shadowing path interactions in three-transducer configurations.
- A shadowing-aware acoustic path selection strategy is proposed to improve wind estimation accuracy by excluding wake-corrupted acoustic paths.

## 2. Materials and Methods

### 2.1. Ultrasonic Anemometer Configuration

**Figure 1:**
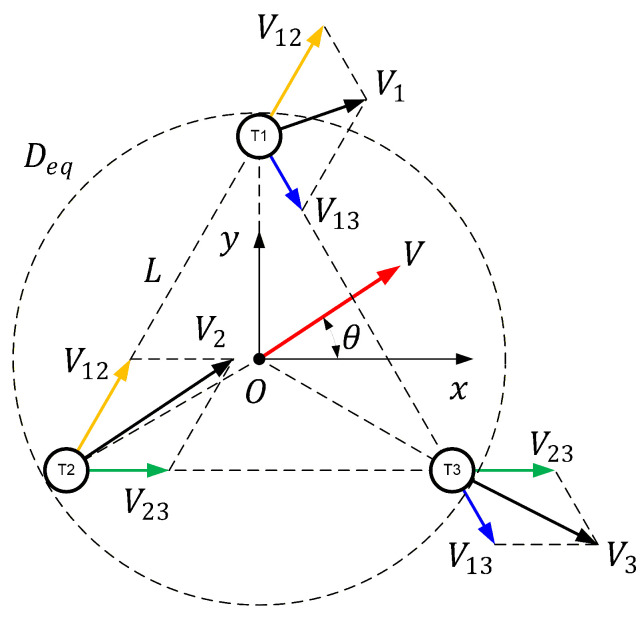
Three-transducer ultrasonic anemometer configuration.

This study considers a three-transducer ultrasonic anemometer arranged symmetrically on a circular frame. The three ultrasonic transducers $T_{1}$, $T_{2}$, and $T_{3}$ are positioned on a circular plane with a diameter of $D_{\mathrm{eq}}=0.285$ m [26] forming an equilateral triangular sensing configuration, as illustrated in Figure 1. Each transducer has a cylindrical structure with radius $r_{\mathrm{trans}}=0.012$ m.

Since ultrasonic pulses are emitted and received from the cylindrical surfaces of the transducers, the effective acoustic propagation path between any two transducers is approximated as the center-to-center distance corrected by the transducer radius, referred to as the line-of-sight (LOS) [27]. Let the horizontal wind velocity vector be denoted by

(1) $$\vec{V}=\left(V,\theta \right)$$

In this expression, *V* represents the wind velocity and θ denotes the wind direction measured with respect to the positive *x*-axis. The wind vector can be projected onto the three acoustic paths connecting the transducer pairs. The resulting LOS velocity components are defined as $V_{12}$, $V_{13}$, and $V_{23}$ corresponding to the acoustic paths between $T_{1}$–$T_{2}$, $T_{1}$–$T_{3}$, and $T_{2}$–$T_{3}$, respectively.

### 2.2. Measurement Principle

Wind velocity is obtained from bidirectional ultrasonic TOF measurements [28,29]. In still air, the propagation time along a path of length *L* is

(2) $$L=c\;t_{0}$$

Here, *c* denotes the velocity of sound. When wind is present, the propagation velocity becomes $c\pm V_{ij}$ depending on the wind direction relative to the acoustic path [30]. For the path between $T_{1}$ and $T_{2}$, the forward and reverse TOF values are

(3) $${\mathrm{TOF}}_{12}=\frac{L}{c+V_{12}}$$

(4) $${\mathrm{TOF}}_{21}=\frac{L}{c-V_{12}}$$

Taking the reciprocal of both expressions and subtracting them eliminates the velocity of sound and isolates the wind-induced velocity component

(5) $$\frac{1}{{\mathrm{TOF}}_{12}}-\frac{1}{{\mathrm{TOF}}_{21}}=\frac{2V_{12}}{L}$$

Thus, the LOS velocity components are obtained as

(6) $$V_{12}=\frac{L}{2}\left(\frac{1}{{\mathrm{TOF}}_{12}}-\frac{1}{{\mathrm{TOF}}_{21}}\right)$$

(7) $$V_{13}=\frac{L}{2}\left(\frac{1}{{\mathrm{TOF}}_{13}}-\frac{1}{{\mathrm{TOF}}_{31}}\right)$$

(8) $$V_{23}=\frac{L}{2}\left(\frac{1}{{\mathrm{TOF}}_{23}}-\frac{1}{{\mathrm{TOF}}_{32}}\right)$$

This formulation effectively removes the influence of temperature-dependent variations in the velocity of sound [28,30]. Due to the symmetric geometry of the sensor, the wind projections along the three paths are

(9) $$\begin{array}{cc} V_{23} & =V\cos\theta \;\;\;\; \end{array}$$

(10) $$\begin{array}{cc} V_{12} & =-V\sin(\theta +30^{\circ })\; \end{array}$$

(11) $$\begin{array}{cc} V_{13} & =V\cos(\theta +60^{\circ })\; \end{array}$$

Using the measured LOS velocity components, three independent estimates of wind velocity and direction can be obtained. For the vertex associated with transducer $T_{1}$, using the pair $\left(V_{12},V_{13}\right)$

(12) $$V_{1}=\frac{2\sqrt{3}}{3}\sqrt{V_{12}^{2}+V_{13}^{2}-V_{12}V_{13}}$$

(13) $$\theta_{1}=\mathrm{atan}2\;\left(\sqrt{3}\left(V_{12}-V_{13}\right),\;V_{12}+V_{13}\right)$$

Using the pairs $\left(V_{12},V_{23}\right)$ and $\left(V_{13},V_{23}\right)$, two additional estimates $\left(V_{2},\theta_{2}\right)$ and $\left(V_{3},\theta_{3}\right)$ are obtained

(14) $$V_{2}=\frac{2\sqrt{3}}{3}\sqrt{V_{23}^{2}+V_{12}^{2}-V_{23}V_{12}}$$

(15) $$\theta_{2}=\mathrm{atan}2\;\left(-\sqrt{3}V_{23},\;2V_{12}+V_{23}\right)$$

(16) $$V_{3}=\frac{2\sqrt{3}}{3}\sqrt{V_{13}^{2}+V_{23}^{2}-V_{13}V_{23}}$$

(17) $$\theta_{3}=\mathrm{atan}2\;\left(-\sqrt{3}V_{23},\;2V_{13}-V_{23}\right)$$

The three redundant estimates provide robustness against measurement errors or flow distortions along individual acoustic paths [14,31,32]. In practice, however, the cylindrical transducers disturb the local airflow and generate wake regions that may intersect with the acoustic paths [33], introducing a systematic bias in the measured LOS velocities and degrading the estimated wind vector. To mitigate this shadowing-induced bias, the direction-dependent selective path-selection strategy is proposed. The algorithm proceeds in two steps. First, each raw direction estimate $\theta_{k}$, obtained from Equations (13), (15) and (17), is converted to the full $\left[0^{\circ },360^{\circ }\right]$ range as follows

(18) $${\tilde{\theta }}_{k}=\left(-\theta_{k}-90^{\circ }\right)\;\mathrm{mod}\;360^{\circ },\;k=1,2,3$$

The wind direction θ is then obtained as the circular mean of the three converted estimates

(19) $$\theta =\mathrm{atan}2\;\left(\sum_{k=1}^{3}\sin{\tilde{\theta }}_{k},\;\sum_{k=1}^{3}\cos{\tilde{\theta }}_{k}\right)\;\mathrm{mod}\;360^{\circ }$$

Second, the wind direction θ is used to identify the corresponding directional sector in Table 1. For each sector, the proposed scheme excludes the wind estimate most susceptible to wake contamination and averages only the remaining redundant estimates to determine the final wind velocity *V*. The selection rules were established by evaluating all possible combinations of $\left(V_{1},V_{2},V_{3}\right)$ over the complete CFD–acoustic simulation dataset and selecting the combination that yielded the minimum RMSE for each directional sector. Consequently, any estimate omitted from the averaging expression is excluded because it experiences the largest wake-induced error in that sector. This behavior is physically explained by the sign-dependent propagation of LOS velocity errors through Equations (12)–(17), whereby wake contamination affects $V_{1}$, $V_{2}$, and $V_{3}$ differently depending on the transducer geometry and wind direction. The resulting selection rules are summarized in Table 1.

## 3. Numerical Simulation

### 3.1. CFD Model

The CFD simulation models the airflow around the three cylindrical transducers and provides the background velocity field used for subsequent acoustic propagation [22,23,34]. The airflow is modeled as an incompressible viscous fluid governed by the Navier–Stokes equations [35]. Under steady-state conditions, the governing equations are

(20) $$\rho \left(u\cdot \nabla \right)u=-\nabla p+\nabla \cdot \left[\left(\mu +\mu_{T}\right)\left(\nabla u+{\left(\nabla u\right)}^{T}\right)\right]$$

(21) ∇·u=0

Here, u is the velocity vector, *p* is the static pressure, ρ is the air density, μ is the dynamic viscosity, and $\mu_{T}$ is the turbulent eddy viscosity. Equation (21) represents the incompressible continuity equation, which enforces mass conservation through a divergence-free velocity field. The incompressible assumption is valid over the simulated velocity range because the maximum freestream velocity of 75m/s corresponds to a Mach number of approximately 0.22, which is below the commonly accepted compressibility limit of Ma=0.3 [36]. In addition, the bidirectional TOF formulation in Equations (3)–(8) removes the dependence on the absolute sound speed through differential measurements, making the wind estimation largely insensitive to variations in the ambient sound speed $c_{0}$ [28,30].

The Reynolds number is defined as

(22) $$Re=\frac{UL}{\nu }$$

where *U* is the freestream wind velocity, *L* is the characteristic length of the sensor geometry, and ν is the kinematic viscosity of air (≈1.5 × 10^−5^ m^2^/s). For the velocity range of 5–75 m/s considered in this study, the Reynolds number ranges from approximately $8\times 10^{3}$ to $1.2\times 10^{5}$, indicating turbulent flow conditions throughout [37].

To capture wake formation and flow separation behind the cylindrical transducers, the standard *k*–ω turbulence model is employed. The turbulent viscosity is computed as

(23) $$\mu_{T}=\rho \frac{k}{\omega }$$

where *k* denotes the turbulent kinetic energy and ω is the specific dissipation rate. The *k*–ω model is selected for its superior treatment of adverse pressure gradients and flow separation in the near-wall region, which are characteristic features of bluff-body wakes [38]. A two-dimensional steady-state formulation was adopted because this study focuses on the mean velocity deficit along the acoustic propagation paths rather than the instantaneous wake structure. The TOF measurement depends on the path-integrated flow field and is therefore primarily affected by the mean wake distortion. Similar steady CFD approaches have been used in previous studies of ultrasonic anemometer shadowing effects [19,24]. In addition, the large span-to-diameter ratio of the cylindrical transducers allows the flow in the acoustic measurement region to be approximated as quasi-two-dimensional.

Appropriate boundary conditions are applied to represent realistic wind flow. The surfaces of the cylindrical transducers are treated as no-slip walls (u=0). The computational domain is fixed for all simulations, while the freestream wind direction θ, defined in Equation (1), is varied from $0^{\circ }$ to $360^{\circ }$. For each wind direction, the prescribed inlet velocity is updated according to the freestream velocity vector:

(24) $$u=U(\cos\theta \;e_{x}+\sin\theta \;e_{y}),$$

At the downstream boundary, a pressure outlet condition is applied, enforced as

(25) $$\left[-pI+K\right]n=-{\hat{p}}_{0}n,\;{\hat{p}}_{0}\leq p_{0}$$

with $K=\left(\mu +\mu_{T}\right)\;\left[\nabla u+{\left(\nabla u\right)}^{T}\right]$, n is the outward unit normal, and the static reference pressure set to $p_{0}=0\;\mathrm{Pa}$ (gauge). The inequality constraint, together with a backflow-suppression condition, restricts this boundary to outflow only. The turbulence quantities satisfy Equation (26), which is consistent with a fully developed outflow.

(26) ∇k·n=0,∇ω·n=0

The path-averaged velocity (path-AV) along each acoustic path is computed as

(27) $${\mathrm{AV}}_{ij}=\frac{1}{L}\int_{0}^{L}\left|u(s)\right|\;ds$$

where $\left|u(s)\right|$ is the local flow speed, and *s* is the arc-length coordinate along the segment connecting transducers $T_{i}$ and $T_{j}$. Path-AV is therefore the mean flow speed along each acoustic path, obtained directly from the CFD velocity field. It is used to quantify the severity of wake-induced flow distortion and identify the acoustic paths most affected by transducer wakes. The resulting path-AV distributions provide the basis for the direction-dependent path-selection strategy proposed in this study. Lower path-AV values indicate stronger wake interference. Conversely, path-AV values greater than the freestream velocity on non-shadowed paths result from local flow acceleration around the cylindrical transducers, which is a well-known characteristic of bluff-body flows [22,23].

The principal numerical settings adopted in the stationary CFD simulation, such as turbulence model, inlet conditions, near-wall treatment, and fluid properties, are summarized in Appendix A (Table A1).

### 3.2. Acoustic Propagation Model

The acoustic simulation is coupled with the background velocity field obtained from the CFD analysis [39,40]. Acoustic propagation is implemented using the Convected Wave Equation interface in COMSOL, which solves a scalar pressure-wave equation in the prescribed background flow. Under the assumption of small-amplitude acoustic perturbations, this formulation is mathematically equivalent to the linearized Euler equations [41]. The background velocity $u_{0}$, pressure $p_{0}$, and density $\rho_{0}$ are imported directly from the stationary CFD solution. Accordingly, the governing equations for the acoustic perturbations are given by

(28) $$\frac{\partial p}{\partial t}+\left(u_{0}\cdot \nabla \right)p+\rho_{0}c_{0}^{2}\nabla \cdot u=0$$

(29) $$\frac{\partial u}{\partial t}+\left(u_{0}\cdot \nabla \right)u+\frac{1}{\rho_{0}}\nabla p=0$$

where $c_{0}\approx 343\;m/s$ is the velocity of sound under standard atmospheric conditions [42]. The velocity field $u_{0}$ is directly obtained from the stationary CFD simulation.

Each transducer alternates between transmitter and receiver roles. The transmitting transducer is modeled as a prescribed normal velocity boundary condition:

(30) $$n\cdot u=v_{n}\left(t\right)$$

A Gaussian-modulated ultrasonic pulse is employed to improve TOF detection in non-uniform flow conditions [43]:

(31) $$v_{n}\left(t\right)=A\;e^{-\frac{{\left(t-t_{0}\right)}^{2}}{2\sigma^{2}}}\sin\left(2\pi f_{0}t\right)$$

where A=1 m/s is the velocity amplitude, $f_{0}=100\;\mathrm{kHz}$ is the transducer operating frequency, $t_{0}=4T_{0}$ is the pulse centre time with $T_{0}=1/f_{0}$, and $\sigma =T_{0}$ controls the temporal width of the Gaussian envelope.

Absorbing boundary conditions are applied at the outer domain to prevent artificial reflections [41]. Rigid-wall conditions enforce zero normal particle velocity at solid surfaces:

(32) n·u=0

The TOF values were extracted by identifying the first carrier-wave pressure peak in the received signal, monitored as the boundary-averaged acoustic pressure over the receiving transducer surface.

### 3.3. Numerical Simulation Setup

**Figure 2:**
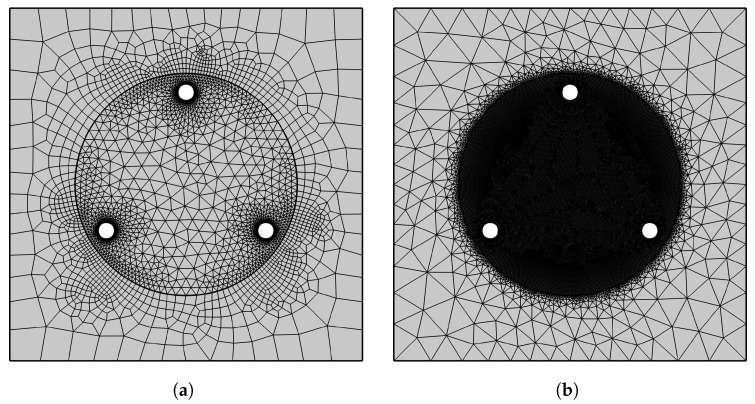
Simulation model mesh: (**a**) CFD model; (**b**) acoustics model.

A square computational domain with a side length of 0.5m was used to approximate open-air conditions. For the present sensor geometry, this corresponds to a per-transducer blockage ratio of approximately 4.8%, which lies within the recommended range for bluff-body wake simulations [44]. The square domain also enables a consistent implementation of the full $360^{\circ }$ wind-direction sweep, while the distance from the sensor center to each lateral boundary exceeds $10D_{\mathrm{trans}}$, satisfying commonly adopted clearance guidelines for cylinder wake simulations [22]. A domain-size sensitivity study confirming that the relative shadowing deficit is independent of the outer-boundary distance is reported in Appendix A (Table A4). To capture the strong velocity gradients near the transducers and within the wake regions, a hybrid mesh was employed, as illustrated in Figure 2. The global computational domain used free quadrilateral elements with maximum and minimum element sizes of 0.0435m and 0.002m, respectively. The mesh growth rate was set to 1.25, the curvature factor to 0.6, and the narrow-region resolution parameter to 1. A refined circular region with a diameter of 0.315m was introduced around the transducers using free triangular elements. Additional local refinement was applied at the transducer boundaries with a maximum element size of 0.014m and a minimum element size of $2\times 10^{-4}\;m$. The corresponding growth rate and curvature factor were set to 1.1 and 0.25, respectively. Corner refinement was applied to regions with angles larger than $240^{\circ }$ using a scaling factor of 0.25. The global boundary-layer configuration consisted of six layers with a stretching factor of 1.2.

The geometry of the acoustic simulation domain was identical to that of the CFD model. The stationary velocity field $u_{0}$ obtained from CFD was imported via multiphysics coupling, with isotropic diffusion (coefficient $10^{-4}$) to reduce interpolation artifacts. Considering $f_{0}=100\;\mathrm{kHz}$, the acoustic wavelength is approximately 3.43mm. The mesh within the inner circular region (diameter 0.315m), which fully encloses the transducer array and all acoustic propagation paths, was refined using free triangular elements with maximum and minimum element sizes of λ/1.5 and λ/2, respectively (growth rate 1.3, curvature factor 0.3). The outer acoustic domain employed a coarser triangular mesh (maximum 0.165m, minimum 0.025m; growth rate 2, curvature factor 1), since it lies outside the region of interest and does not influence the extracted TOF values. This hybrid meshing strategy reduces computational cost and output file size while maintaining full accuracy for acoustic propagation within the transducer array. Four smoothing iterations were applied to ensure smooth mesh transitions. A grid-convergence study verifying that the reported path-averaged velocities are independent of mesh resolution is provided in Appendix A (Table A2).

The CFD simulations were conducted for freestream wind velocities from 5m/s to 75m/s and wind directions from $0^{\circ }$ to $360^{\circ }$. At the same time, the acoustic simulations used a time-dependent solver with a total simulation time of $80T_{0}$, and a time step of $\Delta t=T_{0}/20=0.5\;\mu s$, which also defines the temporal resolution for TOF extraction. The TOF was determined from the first carrier-wave pressure peak in the received signal, providing a velocity resolution well below 0.1m/s, which is negligible compared with the wake-induced velocity deficits investigated in this study.

## 4. Results

### 4.1. CFD Simulation Results

**Figure 3:**
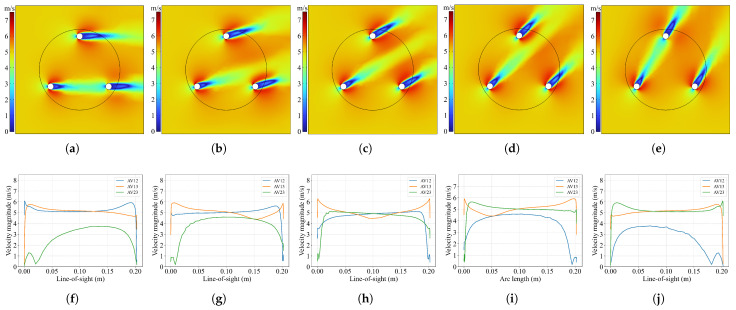
Velocity nephograms and path-averaged velocity magnitude at 5m/s: (**a**) nephogram at $0^{\circ }$; (**b**) nephogram at $15^{\circ }$; (**c**) nephogram at $30^{\circ }$; (**d**) nephogram at $45^{\circ }$; (**e**) nephogram at $60^{\circ }$; (**f**) path-AV at $0^{\circ }$; (**g**) path-AV at $15^{\circ }$; (**h**) path-AV at $30^{\circ }$; (**i**) path-AV at $45^{\circ }$; (**j**) path-AV at $60^{\circ }$.

The velocity fields shown in Figure 3a,e reveal the formation of wake regions downstream of each cylindrical transducer. At a wind direction of $0^{\circ }$, the upstream transducer generates a wake that directly overlaps with the $T_{2}$–$T_{3}$ acoustic path. As a result, the path-AV along this path is significantly reduced to approximately ${\mathrm{AV}}_{23}\approx 2.93\;m/s$, corresponding to a velocity deficit of about 41.4%. In contrast, the remaining acoustic paths experience only minor disturbances, with ${\mathrm{AV}}_{12}\approx 5.26\;m/s$ and ${\mathrm{AV}}_{13}\approx 5.14\;m/s$. The values exceeding the freestream velocity of 5m/s are consistent with local flow acceleration around the upstream transducer body, as discussed in Section 3.1. A quantitative comparison of the theoretical LOS projections and the acoustically inverted TOF values for representative directions ($0^{\circ }$, $30^{\circ }$, $60^{\circ }$) is given in Appendix A (Table A5). Due to the rotational symmetry of the three-transducer configuration, similar wake–path interactions are observed at $60^{\circ }$, as confirmed by Figure 3e,j.

The airflow behavior for intermediate wind directions is illustrated in Figure 3b–d for $15^{\circ }$, $30^{\circ }$, and $45^{\circ }$, respectively. The velocity nephograms demonstrate that the wakes are obliquely oriented relative to the acoustic paths. At $15^{\circ }$, the wake partially intersects one acoustic path, producing ${\mathrm{AV}}_{23}\approx 3.86\;m/s$, corresponding to a velocity deficit of 22.8%. At $30^{\circ }$, the wake shifts further away, yielding a minimum path-AV of approximately 4.76m/s (deviation ≈4.8%). At $45^{\circ }$, the wake begins to re-engage with the $T_{1}$–$T_{2}$ path (Figure 3i), indicating the onset of shadowing as the direction approaches $60^{\circ }$.

**Figure 4:**
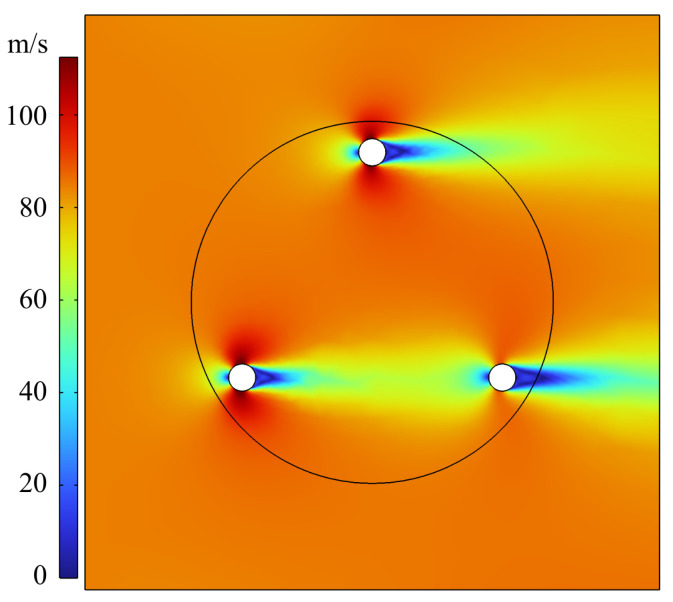
Velocity nephograms at 75m/s with wind direction at $0^{\circ }$.

To investigate the dependence of shadowing on wind velocity magnitude, additional simulations were performed fixing the wind direction at $0^{\circ }$ and increasing the freestream velocity from 5m/s to 75m/s. The velocity nephograms at 75m/s are illustrated in Figure 4. Despite the large increase in freestream velocity, the overall wake structure remains similar. The flow consistently exhibits boundary-layer separation around the cylindrical transducers, followed by a downstream recirculation region and gradual velocity recovery.

**Figure 5:**
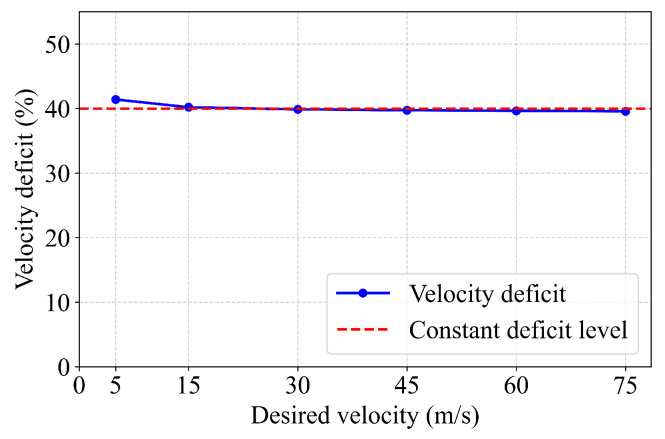
Variation of velocity deficit with freestream velocity.

This observation is quantified in Figure 5, which shows the velocity deficit along the most affected acoustic path for freestream velocities of 5, 15, 30, 45, 60, and 75m/s. The relative velocity reduction remains nearly constant across the entire velocity range, varying only slightly from approximately 39.6% to 41.4%.

**Figure 6:**
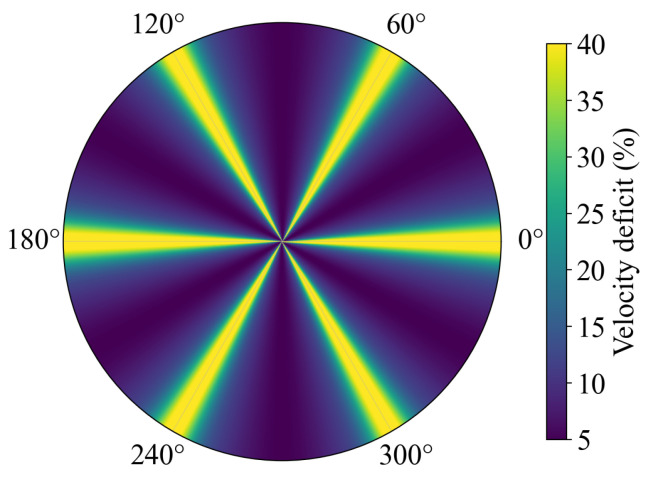
Velocity deficit as a function of wind direction over the full $360^{\circ }$ range.

The directional dependence of the wake-induced distortion, evaluated over the full $360^{\circ }$ range, is summarized in Figure 6. The polar plot reveals a clear periodic pattern consistent with the rotational symmetry of the three-transducer configuration. Within a narrow angular sector of approximately $\pm 5^{\circ }$ around critical directions, the velocity deficit reaches its maximum of approximately 40%. Outside these sectors, the deficit decreases rapidly and is relatively small (around 5%) for most intermediate directions.

### 4.2. Acoustic Propagation Simulation Results

**Figure 7:**
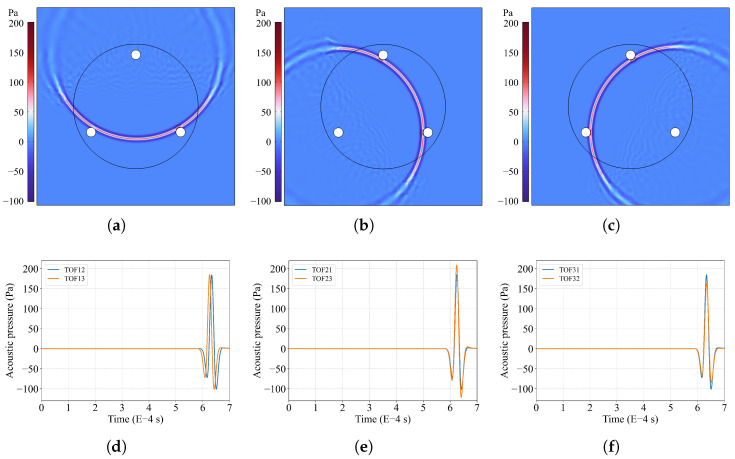
Ultrasonic wave propagation and received signal profiles at 5m/s: (**a**) wave propagation, $T_{1}$ transmitter; (**b**) wave propagation, $T_{2}$ transmitter; (**c**) wave propagation, $T_{3}$ transmitter; (**d**) received signal, $T_{1}$ transmitter; (**e**) received signal, $T_{2}$ transmitter; (**f**) received signal, $T_{3}$ transmitter.

The simulations for transmitters $T_{1}$, $T_{2}$, and $T_{3}$ were conducted independently to prevent signal interference and ensure accurate TOF estimation. As shown in Figure 7a–c, the acoustic wavefronts propagate asymmetrically under airflow influence, reflected in the received signal profiles of Figure 7d–f.

This propagation asymmetry can be quantified by the differential TOF, defined as $\Delta {\mathrm{TOF}}_{ij}=\left|{\mathrm{TOF}}_{ij}-{\mathrm{TOF}}_{ji}\right|$, where ${\mathrm{TOF}}_{ij}$ and ${\mathrm{TOF}}_{ji}$ denote the forward and reverse propagation times along the same acoustic path, respectively. For the $T_{1}$–$T_{3}$ path: ${\mathrm{TOF}}_{13}=6.255\times 10^{-4}\;s$ and ${\mathrm{TOF}}_{31}=6.335\times 10^{-4}\;s$, giving $\Delta {\mathrm{TOF}}_{13}\approx 8.0\times 10^{-6}\;s$. For $T_{1}$–$T_{2}$: ${\mathrm{TOF}}_{12}=6.335\times 10^{-4}\;s$ and ${\mathrm{TOF}}_{21}=6.255\times 10^{-4}\;s$, giving $\Delta {\mathrm{TOF}}_{12}\approx 8.0\times 10^{-6}\;s$. For $T_{2}$–$T_{3}$: ${\mathrm{TOF}}_{23}=6.245\times 10^{-4}\;s$ and ${\mathrm{TOF}}_{32}=6.36\times 10^{-4}\;s$, giving $\Delta {\mathrm{TOF}}_{23}\approx 1.15\times 10^{-5}\;s$.

The $T_{2}$–$T_{3}$ path exhibits the largest ΔTOF, indicating stronger asymmetry due to flow distortion, while the $T_{1}$–$T_{2}$ and $T_{1}$–$T_{3}$ paths have smaller, more comparable values, confirming they are less affected by wake-induced disturbances.

### 4.3. Data Analysis

**Figure 8:**
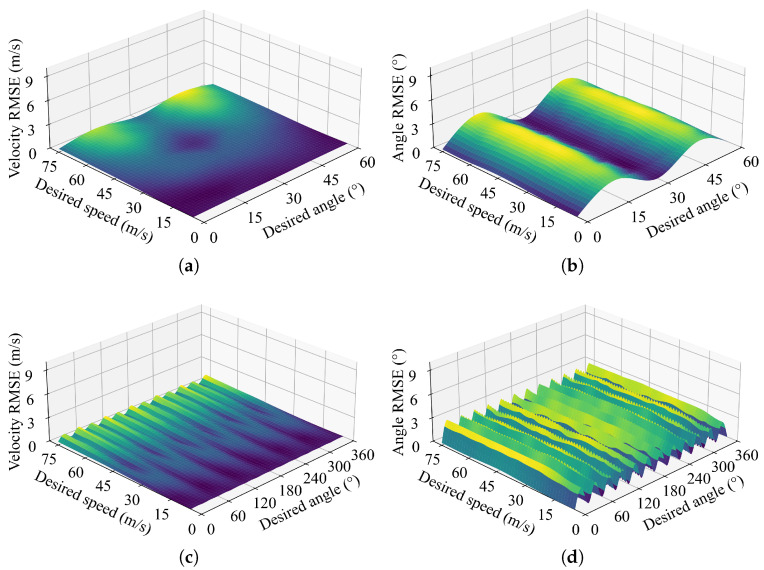
RMSE of estimated wind velocity and direction: (**a**) velocity RMSE, 0–$60^{\circ }$; (**b**) angle RMSE, 0–$60^{\circ }$; (**c**) velocity RMSE, 0–$360^{\circ }$; (**d**) angle RMSE, 0–$360^{\circ }$.

The reconstruction performance was evaluated over the complete CFD–acoustic simulation dataset described in Section 4.1 and Section 4.2. The steady CFD solution and the coupled acoustic simulation are deterministic, so each wind speed–direction case is fully reproducible and yields a single reconstructed wind vector from its bidirectional TOF measurements. The reported RMSE therefore reflects the systematic reconstruction error, obtained by aggregating the deviation of the reconstructed velocity and direction from the reference inputs across the sampled conditions in the dataset. Figure 8a,b present the RMSE distributions of estimated wind velocity and direction for the angular range $0^{\circ }$ to $60^{\circ }$, while Figure 8c,d show the corresponding results over the full $360^{\circ }$ range.

At lower wind velocities, the estimated values closely match the reference inputs. The minimum velocity RMSE is approximately 0.015m/s. As freestream velocity increases, the RMSE rises gradually but the proposed method maintains stable performance. The velocity RMSE increases from approximately 0.051m/s at 5m/s to 0.763m/s at 75m/s, with an overall mean of 0.349m/s; even at the highest velocity the RMSE remains below 1.3m/s. The direction error exhibits a periodic dependence on wind direction. The smallest errors occur near $0^{\circ }$, $30^{\circ }$, and $60^{\circ }$; in particular, $30^{\circ }$ yields near-zero directional RMSE across all simulated velocities. The largest directional errors occur at $15^{\circ }$ and $45^{\circ }$, with a maximum angular RMSE of approximately $2.89^{\circ }$ at $15^{\circ }$. Despite these localized peaks, the overall average directional RMSE is approximately $1.14^{\circ }$.

To further assess the effectiveness of the proposed shadow-aware estimation strategy, its performance was compared with a shadow-unaware baseline that reconstructs the wind velocity using the conventional uniform average $(V_{1}+V_{2}+V_{3})/3$ without excluding wake-corrupted LOS measurements [19,32].

**Figure 9:**
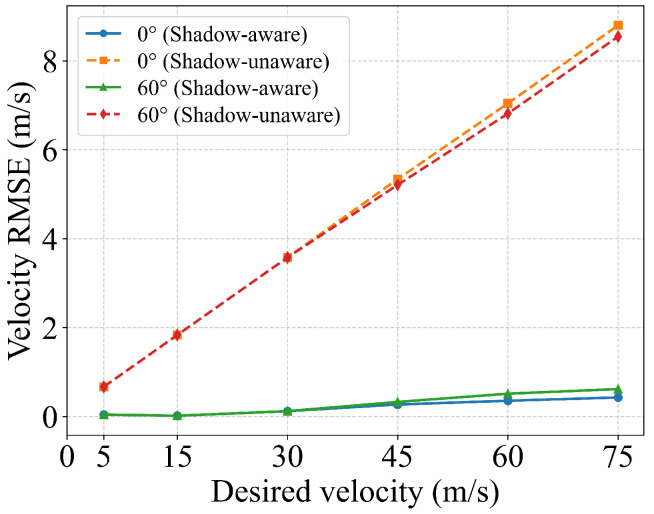
Comparison of velocity RMSE between shadow-aware and shadow-unaware methods at wind directions of $0^{\circ }$ and $60^{\circ }$.

Figure 9 compares the two methods at wind directions of $0^{\circ }$ and $60^{\circ }$. The shadow-unaware baseline produces RMSE that grows steadily with wind speed, from 0.67m/s at 5m/s to about 8.55–8.80m/s at 75m/s. The proposed shadow-aware method keeps the RMSE far lower across the same range, from 0.02m/s at 5m/s to 0.43–0.62m/s at 75m/s, an improvement of roughly 14 to 21 times over the shadow-unaware baseline at the highest tested speed.

**Figure 10:**
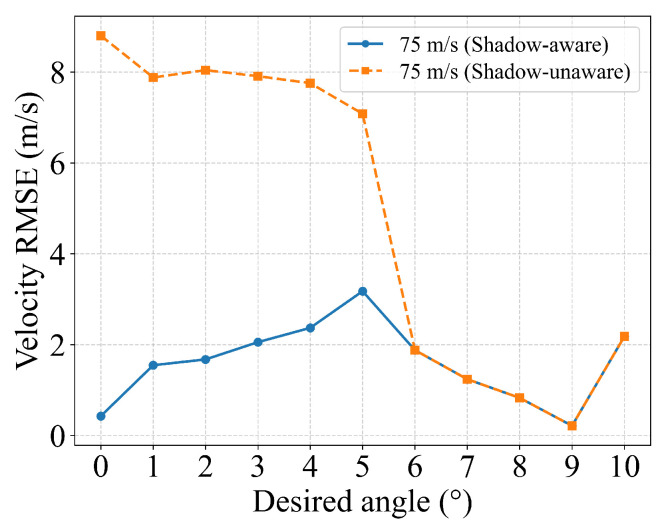
Comparison of velocity RMSE between shadow-aware and shadow-unaware methods at 75m/s for small wind direction angles.

Additional simulations at 75m/s with finer angular resolution between $0^{\circ }$ and $10^{\circ }$ (Figure 10) reveal that severe errors from the shadow-unaware method are confined primarily to the 0–$5^{\circ }$ sector (RMSE between 7 and 9m/s). The proposed shadowing-aware acoustic path selection method reduces RMSE significantly across this range, with the most notable improvement near $0^{\circ }$. Beyond approximately $6^{\circ }$, both methods converge to RMSE values below 2m/s.

## 5. Discussion

The simulation results confirm that aerodynamic disturbances generated by the cylindrical transducers introduce wake regions that significantly affect ultrasonic wind measurements. When these wakes intersect an acoustic propagation path, the resulting velocity deficit alters the effective flow velocity experienced by the ultrasonic wave, producing systematic deviations in the measured TOF. Compared with the CFD-based investigation reported by Chen et al. [19], which analyzed transducer shadowing only up to approximately 13.45m/s and reported velocity errors below 0.5m/s within that limited range, the present study extends the analysis to 75m/s and demonstrates that estimation error increases steadily when shadowing is not corrected, reaching up to 8.80m/s RMSE at a desired velocity of 75m/s under severe shadowing conditions. Li et al. [24] similarly confined their PIV-CFD analysis to 20m/s, reporting average relative velocity errors of 3.38% and peak errors of 4.25%, leaving the shadowing behavior at higher wind speeds uncharacterized. Zhao et al. [18] proposed a phase-difference-based cross-correlation algorithm operating at wind speeds up to 12m/s; however, the absence of CFD-based aerodynamic modeling means that shadowing-induced bias was not accounted for, and the reported accuracy of approximately 0.2m/s in velocity and $1^{\circ }$ in direction does not reflect physically realistic flow conditions. Similarly, while Shan et al. [32] employs a three-sensor TOF-based estimation framework, the absence of CFD-based airflow modeling means that shadowing-induced bias is not captured, and the reported errors reflect idealized rather than physically realistic conditions. By integrating CFD flow analysis with acoustic simulation and a direction-dependent acoustic path selection strategy, this work provides an explicit, quantitative bridge between idealized simulation assumptions and the aerodynamic realities of three-transducer configurations.

## 6. Conclusions

This paper presented a comprehensive CFD-based analysis and a shadowing-aware path-selection method for a three-transducer ultrasonic anemometer. The proposed approach identifies and excludes acoustic paths corrupted by transducer wakes from the wind estimation process, thereby improving wind estimation accuracy through selective path usage. By coupling CFD-based airflow simulation with acoustic propagation modeling, the interaction between wake-induced velocity deficits and TOF measurement errors was characterized across a wide operational range. The results demonstrate that shadowing-induced distortions are strongly direction-dependent, peaked within approximately $\pm 5^{\circ }$ angular sectors, and governed primarily by sensor geometry rather than wind speed, with a near-constant velocity deficit of approximately 40% along affected acoustic paths.

Based on these findings, a direction-dependent wind estimation selection method was proposed that systematically omits corrupted acoustic paths from the estimation process. The approach achieves an average velocity RMSE of 0.349m/s and a directional RMSE of $1.14^{\circ }$ across wind velocities from 5 to 75m/s over the full $360^{\circ }$ directional range. Future work will first validate the proposed method through controlled wind-tunnel experiments and then extend the numerical model to incorporate unsteady vortex-shedding effects on TOF jitter, three-dimensional wake effects, ambient turbulence, and temperature variations.

## Figures and Tables

**Table 1:** Wind velocity selection rules based on the wind direction θ.

Wind Direction θ	Wind Velocity *V*
$0^{\circ }\leq \theta \leq 5^{\circ }$	$(V_{1}+V_{2})/2$
$5^{\circ }<\theta <55^{\circ }$	$(V_{1}+V_{2}+V_{3})/3$
$55^{\circ }\leq \theta \leq 65^{\circ }$	$(V_{2}+V_{3})/2$
$65^{\circ }<\theta <115^{\circ }$	$(V_{1}+V_{2}+V_{3})/3$
$115^{\circ }\leq \theta \leq 125^{\circ }$	$(V_{1}+V_{3})/2$
$125^{\circ }<\theta <175^{\circ }$	$(V_{1}+V_{2}+V_{3})/3$
$175^{\circ }\leq \theta \leq 185^{\circ }$	$(V_{1}+V_{2})/2$
$185^{\circ }<\theta <235^{\circ }$	$(V_{1}+V_{2}+V_{3})/3$
$235^{\circ }\leq \theta \leq 245^{\circ }$	$(V_{2}+V_{3})/2$
$245^{\circ }<\theta <295^{\circ }$	$(V_{1}+V_{2}+V_{3})/3$
$295^{\circ }\leq \theta \leq 305^{\circ }$	$(V_{1}+V_{3})/2$
$305^{\circ }<\theta <355^{\circ }$	$(V_{1}+V_{2}+V_{3})/3$
$355^{\circ }\leq \theta <360^{\circ }$	$(V_{1}+V_{2})/2$

## Data Availability

The original contributions presented in this study are included in the article. Further inquiries can be directed to the corresponding authors.

## Abbreviations

The following abbreviations are used in this manuscript:

TOF	Time-of-flight
CFD	Computational fluid dynamics
LOS	Line-of-sight
RMSE	Root-mean-square error
PIV	Particle Image Velocimetry
UAV	Unmanned aerial vehicle
URWS	Ultrasonic resonance wind sensing
path-AV	Path-averaged velocity

## Appendix A

This appendix collects the CFD solver settings and the supporting numerical-validation studies referenced in the main text. Table A1 lists the principal CFD solver settings. Table A2, Table A3 and Table A4 report the grid-convergence and domain-size sensitivity studies used to establish the numerical reliability of the simulation. Table A5 compares the theoretical LOS projections with the acoustically inverted TOF values for representative wind directions.

Table A1 summarizes the turbulence model, inlet conditions, near-wall treatment, and fluid properties adopted throughout the stationary CFD simulation.

A grid-convergence study was performed for the critical case $\theta =0^{\circ }$, V=5m/s, in which the $T_{2}$–$T_{3}$ path is fully shadowed, and the near-wall velocity gradients are most severe. Three mesh levels were generated using the calibrated automatic presets of COMSOL Multiphysics (Coarser, Normal, and Fine), yielding 2768, 3887, and 3941 domain elements, respectively. The path-averaged velocity on each acoustic path was used as the convergence metric, as summarized in Table A2. The two unshadowed paths vary by less than 0.07% across the full series. In comparison, the shadow-affected path ${\mathrm{AV}}_{23}$ varies by less than 0.1% overall and by only 0.03% between the Normal and Fine levels, so that the resulting velocity deficit remains within 41.33–41.37%. The solution is therefore grid-independent, and the Coarser preset, already within the converged regime, was adopted for the full production sweep to minimize computational cost over the large parametric space.

**Table A1:** Summary of the CFD simulation setup.

Parameter	Value
Flow model	Standard *k*–ω RANS
Wall treatment	Automatic wall treatment
Wind speed range	5–75m/s
Inlet turbulence intensity	5%
Inlet turbulence length scale	$8.4\times 10^{-4}\;m$
First-layer $y^{+}$ range	≈5 (at 5m/s) to 59 (at 75m/s)
Reference temperature	293.15K
Air density	$1.204\;\mathrm{kg}/m^{3}$
Dynamic viscosity	$1.825\times 10^{-5}\;\mathrm{Pa}\cdot s$

**Table A2:** Grid-convergence study at $\theta =0^{\circ }$, V=5m/s.

Mesh	Elements	${\mathrm{AV}}_{12}$ (m/s)	${\mathrm{AV}}_{13}$ (m/s)	${\mathrm{AV}}_{23}$ (m/s)
Coarser	2768	5.2580	5.1362	2.9334
Normal	3887	5.2562	5.1329	2.9315
Fine	3941	5.2567	5.1338	2.9324

A second grid-convergence study was also carried out at $\theta =0^{\circ }$ and V=75m/s, representing the highest simulated wind velocity. The results are listed in Table A3. Variations in path-AV are below 0.2% for the unshadowed paths and below 0.06% for the shadowed path, demonstrating that the selected mesh remains grid-independent throughout the simulated velocity range.

**Table A3:** Grid-convergence study at $\theta =0^{\circ }$, V=75m/s.

Mesh	Elements	${\mathrm{AV}}_{12}$ (m/s)	${\mathrm{AV}}_{13}$ (m/s)	${\mathrm{AV}}_{23}$ (m/s)
Coarser	2768	78.831	77.169	42.581
Normal	3887	78.752	77.033	43.224
Fine	3941	78.772	77.042	43.248

A domain-size sensitivity study was conducted for the same critical case at three square-domain side lengths (0.5, 1.0, and 1.5m), as reported in Table A4. All path-averaged velocities decrease uniformly by approximately 1.5% per step, a mild blockage effect consistent with the confined domain. Critically, the ratio ${\mathrm{AV}}_{23}/{\mathrm{AV}}_{12}$ between the shadowed and unshadowed paths remains essentially constant (varying by less than 0.2% from 0.5 to 1.5m). Since it is this relative deficit, not the absolute path-averaged magnitude, that governs the shadow-induced TOF bias and the reconstruction error, the results are domain-independent with respect to the quantity of interest, and the 0.5m production domain is adequate.

**Table A4:** Domain-size sensitivity study at $\theta =0^{\circ }$, V=5m/s.

Domain Side (m)	${\mathrm{AV}}_{12}$ (m/s)	${\mathrm{AV}}_{13}$ (m/s)	${\mathrm{AV}}_{23}$ (m/s)	${\mathrm{AV}}_{23}/{\mathrm{AV}}_{12}$
0.5	5.261	5.142	2.931	0.557
1.0	5.186	5.067	2.885	0.556
1.5	5.109	4.990	2.841	0.556

Table A5 distinguishes the CFD path-averaged velocity magnitude |u| (used only to visualize wake severity) from the LOS-projected velocity that enters the TOF formulation. For each representative direction, it lists the theoretical unshadowed LOS projection $V_{ij}$ (theory), the acoustically inverted value $V_{ij}$ (TOF), and the relative error. The deviations correspond to three identifiable aerodynamic mechanisms: the downstream wake shadow (≈41% on the fully shadowed path), the flow acceleration/deceleration near the receiving transducer (≈6.8% on the adjacent paths), and the upstream blockage effect (≈19.1% at intermediate directions).

**Table A5:** Validation of the LOS-projected velocity against the acoustically inverted TOF values at V=5m/s.

Direction	Path	${\mathrm{AV}}_{\mathrm{ij}}$ (CFD, |u|)	$V_{\mathrm{ij}}$ (Theory)	$V_{\mathrm{ij}}$ (TOF)	|Error| (%)
$0^{\circ }$ ($V_{23}$ shadowed)	$V_{12}$	5.261	−2.5	−2.331	6.8
	$V_{13}$	5.142	2.5	2.331	6.8
	$V_{23}$	2.931	5.0	2.914	41.4
$30^{\circ }$ (mild distortion)	$V_{12}$	4.645	−4.330	−3.502	19.1
	$V_{13}$	5.089	0	0	—
	$V_{23}$	4.640	4.330	3.502	19.1
$60^{\circ }$ ($V_{12}$ shadowed)	$V_{12}$	2.979	−5.0	−2.914	40.4
	$V_{13}$	5.148	−2.5	−2.331	6.8
	$V_{23}$	5.261	2.5	2.331	6.8
